# Pengzhenrongella frigida sp. nov., isolated from a glacier

**DOI:** 10.1099/ijsem.0.006433

**Published:** 2024-06-19

**Authors:** Qing Liu, Lei-Lei Yang, Yu-Hua Xin

**Affiliations:** 1 China General Microbiological Culture Collection Center, Institute of Microbiology, Chinese Academy of Sciences, Beijing 100101, PR China

**Keywords:** cold-adapted, glacier, *Pengzhenrongella*, *Pengzhenrongella frigida*

## Abstract

A Gram-stain-positive, rod-shaped bacterium, designated as HLT2-17^T^, was isolated from soil sample taken from the Hailuogou glacier in Sichuan province, PR China. Strain HLT2-17^T^ was capable of growing at 4–25°C and in NaCl concentrations ranging from 0 to 2% (w/v). The highest level of 16S rRNA gene sequence similarity was observed with *Pengzhenrongella phosphoraccumulans* M0-14^T^ (98.3 %) and *Pengzhenrongella sicca* LRZ-2^T^ (98.2 %). The average nucleotide identity and digital DNA–DNA hybridization values between strain HLT2-17^T^ and its closest relatives, *P. phosphoraccumulans* M0-14^T^ and *P. sicca* LRZ-2^T^, were 80.0–84.0 % and 23.3–27.7 %, respectively. Phylogenomic analysis indicated that strain HLT2-17^T^ clustered together with strains *P. phosphoraccumulans* M0-14^T^ and *P. sicca* LRZ-2^T^. Strain HLT2-17^T^ contained C_16 : 0_ and anteiso-C_15 : 0_ as the major fatty acids, and MK-9(H_4_) as the menaquinone. Therefore, based on a polyphasic approach, we propose that strain HLT2-17^T^ (=CGMCC 1.11116^T^= NBRC 110443^T^) represents a novel species of the genus *Pengzhenrongella* and suggest the name *Pengzhenrongella frigida* sp. nov.

## Introduction

The genus *Pengzhenrongella*, a member of the suborder *Micrococcineae* within the order *Actinomycetales*, was first established by Kim *et al*. in 2021 with *Pengzhenrongella sicca* as the type species [1]. Members of this genus are Gram-stain-positive, aerobic, and non-sporulating, with a high DNA G+C content (72.4 mol%). Bacteria of this genus contain MK-9 (H_4_) as the major menaquinone, and have anteiso-C_15 : 0_ and iso-C_16 : 0_ as the major fatty acids [1]. At the time of writing, this genus comprises only two species with validly published and correct names [2]. The type strains of *Pengzhenrongella sicca* [1] and *Pengzhenrongella phosphoraccumulans* [3] was isolated from a soil sample collected from the High Arctic tundra [1]. During a comprehensive survey of bacterial diversity on the surfaces of glaciers in PR China, we isolated a significant number of bacterial strains. Among them, a strain representing a novel species belonging to the genus *Pengzhenrongella* was identified. This strain was isolated from a soil sample taken from the Hailuogou glacier situated in Sichuan province, PR China. This paper will be the third report of the genus *Pengzhenrongella* found in the cryosphere, which will enhance our understanding of the distribution of this genus in cold environments. In addition, a robust phylogenomic tree of the family *Cellulomonadaceae* was reconstructed using genomic sequences.

## Isolation and ecology

Soil samples collected from the surface of the Hailuogou glacier (101.97° E, 29.56° N) were homogenized and diluted in series with sterile water. After a 20 days’ incubation on Reasoner’s 2A (R2A) agar (BD Difco) at 15°C, the isolates were picked up and purified. A yellow-coloured colony, designated as HLT2-17^T^, was selected and subjected to further study. Strain HLT2-17^T^ was preserved in aqueous glycerol suspensions (10 %, v/v) in a liquid nitrogen storage tank and routinely incubated in R2A medium at 20°C. Type strains of *Cellulomonas aerilata* 5420 S-23^T^ (=CGMCC 4.7075^T^) and *Cellulomonas biazotea* 127^T^ (=CGMCC 1.1899^T^) obtained from the China General Microbiological Culture Collection Center (CGMCC), were used as experimental controls.

## 16S rRNA phylogeny

The genomic DNA was extracted with TaKaRa MiniBEST Bacteria Genomic DNA Extraction Kit version 3.0 following the manufacturer’s instructions. The 16S rRNA gene was amplified and sequenced using the universal primer pairs 27F and 1492R [4]. The 16S rRNA gene sequence of strain HLT2-17^T^ was submitted to EzBioCloud to search for related species [5]. Multiple sequences were aligned with the ClustalW program implemented in mega version 5.2 [6]. Neighbour-joining (NJ), maximum-likelihood (ML), and maximum-parsimony (MP) phylogenetic trees were reconstructed and evaluated using mega version 5.2. Kimura’s two parameter model was used to calculate the genetic distances for the NJ analysis. The tree topologies were evaluated by the bootstrap values based on 1000 resamplings.

The 16S rRNA gene sequences revealed that strain HLT2-17^T^ belonged to the genus *Pengzhenrongella* and exhibited the highest sequence similarity to *P. phosphoraccumulans* M0-14^T^ (98.3 %) and *P. sicca* LRZ-2^T^ (98.2 %). The similarities between strain HLT2-17^T^ and its closest neighbours were below the 98.65 % similarity threshold for species distinction suggested by Kim *et al*. [7], indicating that strain HLT2-17^T^ represented a novel species in the genus *Pengzhenrongella*. Strain HLT2-17^T^ clustered together with its closest relatives, *P. phosphoraccumulans* M0-14^T^ and *P. sicca* LRZ-2^T^, in the NJ (Fig. S1, available in the online version of this article), ML (Fig. S2) and MP (Fig. S3) phylogenetic trees.

## Genome features

The whole genome sequencing of strain HLT2-17^T^ was carried out with the Illumina HiSeq 4000 platform with 150 bp paired-end reads according to the manufacturer’s protocols. Short reads were assembled *de novo* using the SPAdes version 3.15 program [8]. The CheckM2 version 1.0.2 program [9] was used to check the completeness and contamination values of the genomes. The genome sequences were functionally annotated using Prokka version 1.13 software [10]. The complete 16S rRNA gene sequence was also retrieved from the genome sequences by Prokka. The natural product biosynthetic gene clusters were identified using antiSMASH version 7.0 [11]. The BacMet database 2.0 was utilized for the functional annotation of antibacterial biocide and metal-resistance genes [12]. The digital DNA–DNA hybridization (dDDH) values were determined using the TYGS as implemented on the DSMZ website [13]. The pairwise average nucleotide identity (ANI) values were calculated using FastANI version 1.33 [14]. A total of 92 core genes were extracted by the UBCG program [15] from genomic sequences and the alignments were generated using mafft version 7.520 software [16]. An ML phylogenetic tree was generated using iq-tree version 2.0.7 [17] based on the concatenated core gene sequences with 1000 bootstrap replicates, with the best nucleotide substitution model of GTR+F+R6.

The completeness of the genome sequence of strain HLT2-17^T^ was about 99.94% with 0.13% contamination, which was inferred by the CheckM2 program. The assembled draft genome of strain HLT2-17^T^ contained 126 contigs, with a total length of 4.32 Mbp and N50 length of 71 287 bp. The genomic DNA G+C content was calculated to be 71.6 mol%. There were 3928 genes, 3873 protein-coding genes and 55 RNA loci (tRNA, 51; 16S rRNA, 1; 23S rRNA, 1; 5S rRNA, 1; tmRNA, 1) predicted using Prokka.

As shown in Table S1, 3738 protein-coding sequences of strain HLT2-17^T^ were classified into COG categories. More than 5% genes of strain HLT2-17^T^ were involved in various functions, including ‘carbohydrate transport and metabolism’ (422), ‘transcription’ (320), ‘general function prediction only’ (299), ‘signal transduction mechanisms’ (296), ‘amino acid transport and metabolism’ (249), ‘coenzyme transport and metabolism’ (227), ‘cell wall/membrane/envelope biogenesis’ (221), ‘translation, ribosomal structure and biogenesis’ (215), ‘inorganic ion transport and metabolism’ (214), ‘energy production and conversion’ (209), and ‘posttranslational modification, protein turnover, chaperones’ (158). Additionally, the gene clusters responsible for synthesis of secondary metabolites were also analysed. A total of 126 genes were identified to be associated with five clusters, including two types of terpene, one lassopeptide, one type III polyketide synthases (T3PKS), and one non-alpha poly-amino acids like e-polylysin. Notably, the T3PKS cluster was found to exhibit 100% similarity to the alkylresorcinol biosynthetic gene cluster from *Streptomyces griseus* subsp. *griseus*. Four genes were annotated as *fabL*/*ygaA*, *fetA*/*ybbL*, *arsC*, and *tupC*, indicating that strain HLT2-17^T^ has the potential ability to resist triclosan, H_2_O_2_, arsenic, and tungsten.

The complete 16S rRNA gene sequence (1 524 bp) of strain HLT2-17^T^, which was retrieved from the genome sequence, was identical to that generated from the PCR product. The ANI and dDDH values between strain HLT2-17^T^ and *P. phosphoraccumulans* M0-14^T^, as well as *P. sicca* LRZ-2^T^ ranged from 80.0 to 84.0% and from 23.3 to 27.7 %, respectively. Additionally, the ANI and dDDH values between strain HLT2-17^T^ and the members of the genus *Cellulomonas* were below 80.2 and 21.7 %, respectively. These values are far below the commonly accepted species delineation cutoffs of 95–96 % for ANI and 70 % for dDDH [18,19], indicating that strain HLT2-17^T^ represents a novel species.

**Fig. 1.**
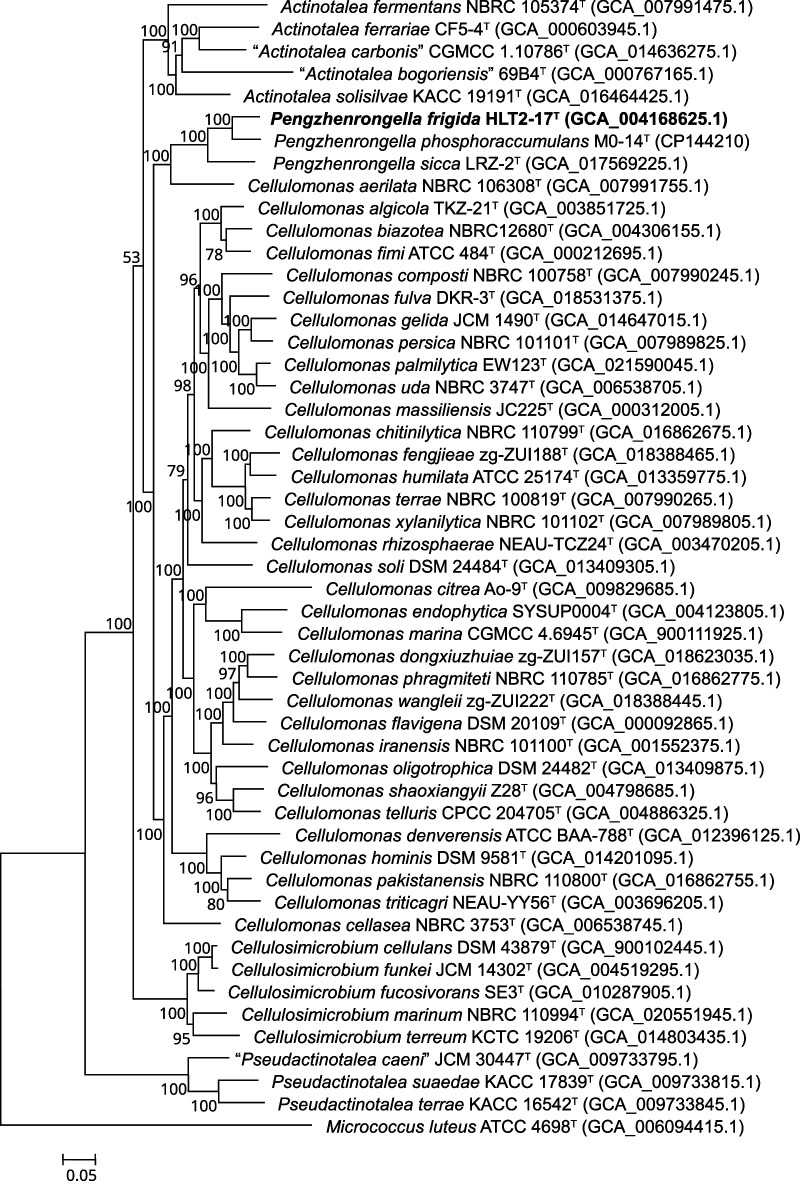
Phylogenomic tree showing the relationships between strain HLT2-17^T^ and its related species, reconstructed based on concatenated alignment of 92 core genes using the nucleotide substitution model of GTR+F+I+G4. Bootstrap values are shown at branching points. Bar, 0.05 substitutions per nucleotide positions.

To further determine the taxonomic status of strain HLT2-17^T^, a robust phylogenetic tree was reconstructed using genomic sequences, encompassing all species of the family *Cellulomonadaceae* (Fig. 1). The phylogenomic tree clearly indicated that strain HLT2-17^T^ belonged to the genus *Pengzhenrongella* and formed a distinct branch with *P. phosphoraccumulans* M0-14^T^ and *P. sicca* LRZ-2^T^. However, the genus *Cellulomonas* appeared non-monophyletic in the phylogenomic tree, suggesting a discrepancy between the classification and phylogeny of the genus. Most *Cellulomonas* species clustered together in one clade, while *C. aerilata* NBRC 106308^T^ formed a separate clade with the genus *Pengzhenrongella*.

## Physiology and chemotaxonomy

Cellular morphology of strain HLT2-17^T^ was examined by transmission electron microscopy (JEM-1400, jeol). Motility was observed by phase-contrast microscopy using the hanging drop method. The growth temperature range test was carried out at 4, 10, 15, 20, 25, 30, 31, and 32°C in R2A broth. The R2A broth for testing growth pH range was adjusted from pH 5.0 to pH 10.0 (at intervals of 1.0 pH unit) with biological buffers (Na_2_HPO_4_/NaH_2_PO_4_ for pH 5–8 and Na_2_CO_3_/NaHCO_3_ for pH 9–10) and then filter-sterilized. Tolerance of NaCl was determined in R2A broth containing various concentrations of NaCl (0–3 % w/v) at intervals of 1.0%. Hydrolysis of casein, starch, and Tween 80 was tested according to Smibert and Krieg [20]. Carbon source utilization was tested using the Biolog GEN III Microstation. Enzyme activities and other biochemical characteristics were performed using the API 20E, 20NE and ZYM strips (bioMérieux) according to the manufacturer’s instructions. The whole-cell fatty acids of strain HLT2-17^T^ and the reference strains were extracted, methylated according to Sasser [21], and analysed by Agilent 6890 gas chromatograph (ultra-2) using the Sherlock Microbial Identification System (version 6.0). Polar lipids were examined with TLC plates (silica gel 60 aluminum-backed, Merck 1.05553) according to Komagata and Suzuki [22]. The menaquinones were determined as described by Collins [23] and analysed by HPLC using a YMC-Pack ODS-A column (150×4.6 mm^2^) with elution of methanol–isopropyl ether (3 : 1, v/v) at a flow rate of 1 ml min^−1^, followed by the detection of ultraviolet absorbance at the wavelength of 275 nm.

Cells of strain HLT2-17^T^ were short rods with flagella (Fig. S4). The temperature range for growth was 4–30°C and optimal growth occurred at 20°C. The pH range for growth was pH 7.0–10.0. NaCl tolerance was up to 2% (w/v). Cells of strain HLT2-17^T^ were positive for catalase and hydrolysis of starch. Strain HLT2-17^T^ were positive for nitrate reduction, hydrolysis of aesculin, Voges–Proskauer test, *β*-galactosidase, *α*-glucosidase, and *β*-glucosidase. Negative for arginine dihydrolase, lysine decarboxylase, ornithine decarboxylase, citrate utilization, urease, H_2_S, and indole production, tryptophane deaminase, alkaline phosphatase, esterase (C4), esterase lipase (C8), lipase (C14), leucine arylamidase, valine arylamidase, cystine arylamidase, trypsin, *α*-chymotrypsin, acid phosphatase, *α*-galactosidase, *β*-glucuronidase, naphthol-AS-BI-phosphohydrolase, *α*-mannosidase, *α*-fucosidase, and *N*-acetyl-*β*-glucosaminidase. The distinguishing phenotypic characteristics between strain HLT2-17^T^ and the reference strains are listed in Table 1. The whole-cell fatty acid compositions of strain HLT2-17^T^ and the reference strains are listed in Table S2. The main fatty acids (>5 %) of strain HLT2-17^T^ were anteiso-C_15 : 0_ (36.6%), C_16 : 0_ (15.4%), summed feature 3 (8.4 %), anteiso-C_15 : 1_ A (6.8%), iso-C_14 : 0_ (6.2%), iso-C_16 : 0_ (5.3%). Strain HLT2-17^T^ and *P. phosphoraccumulans* M0-14^T^ [3] both contained anteiso-C_15 : 0_ and C_16 : 0_ as the major fatty acid. In contrast, *P. sicca* LRZ-2^T^ contained more anteiso-C_15 : 1_ [1]. The polar lipids detected in strain HLT2-17^T^ were diphosphatidylglycerol (DPG), phosphatidylinositol (PI), phosphatidylinositol mannoside (PIM), one unidentified phosphoglycolipid and two unidentified lipids (Fig. S5). There is a significant difference in polar lipid composition between strain HLT2-17^T^ and its closest relative, *P. phosphoraccumulans* M0-14^T^ (Table 1). For example, strain HLT2-17^T^ contained DPG, which was absent in *P. phosphoraccumulans* M0-14^T^ [3]. In addition, *P. phosphoraccumulans* M0-14^T^ had phosphatidylglycerol, which was absent in strain HLT2-17^T^. The sole menaquinone is MK-9 (H_4_), which was consistent with the characteristics of *Pengzhenrongella* species [1,3].

**Table 1.** Phenotypic characteristics differentiating strain HLT2-17^T^ from type strains of related species Strains: 1*,* HLT2-17^T^; 2, *Pengzhenrongella phosphoraccumulans* M0-14^T^; 3, *Pengzhenrongella sicca* LRZ-2^T^; 4, *Cellulomonas biazotea* CGMCC 1.1899^T^; 5, *Cellulomonas aerilata* CGMCC 4.7075^T^. +, Positive, –, negative; na, not available.

Characteristic	1	2*	3*	4	5
Optimum temperature for growth (°C)	20	4–18	18–22	28	28
NaCl range for growth (%, w/v)	0–2.0	0–5.0	0–3.0	0–2.5	0–1.0
Motility	+	+	–	+	+
Hydrolysis of gelatin	–	–	+	+	–
Enzymatic activities:					
Urease	–	–	–	–	+
Alkaline phosphatase	–	–	–	+	+
Leucine arylamidase	–	+	+	+	+
*β-*Galactosidase	+	+	+	+	–
*β*-Glucosidase	+	+	+	+	–
*N*-Acetyl-*β*-glucosaminidase	–	–	+	+	+
Major polar lipids†	DPG, PI, PIM, PGL	PG, PI, PIM	DPG, PI, PIM, PGL	na	PG, DPG*
G+C content (mol%)	71.6	70.8	72.4	74.5	74.4

*Data from Xie *et al*. [3], Kim *et al*. [1], and Lee *et al*. [24].

†DPG, diphosphatidylglycerol; PG, phosphatidylglycerol; PI, phosphatidylinositol; PIM, phosphatidy linositol mannoside; PGL, unidentified phosphoglycolipid.

In conclusion, on the basis of phenotypic, phylogenetic and genotypic characteristics, we conclude that strain HLT2-17^T^ represents a novel species of the genus *Pengzhenrongella*, for which the name *Pengzhenrongella frigida* sp. nov. is proposed.

## Description of *Pengzhenrongella frigida* sp. nov.

*Pengzhenrongella frigida* (fri'gi.da. L. fem. adj. *frigida*, cold; referring to the cold habitat from which the type strain was isolated).

Cells are aerobic, Gram-stain-positive, motile with flagella, short-rod-shaped, 0.5–0.7 µm wide, and 0.8–1.6 µm long. Colonies are light-yellow-pigmented, circular, smooth, convex, and 1.0 mm in diameter after 7 days incubation on R2A plates at 20°C. Growth occurs at 4–25°C, at pH 7.0–10.0 and in the presence of 0–2 % (w/v) NaCl. Catalase activity is observed, but oxidase activity is absent. Hydrolyses starch, but does not hydrolyse casein, gelatin and Tween 80. Cells are positive for nitrate reduction, hydrolysis of aesculin, and the Voges–Proskauer test. Negative for citrate utilization, urease, H_2_S and indole production. Acid is produced from d-glucose, l-rhamnose, sucrose, amygdalin and l-arabinose, but not from d-manitol, inositol, d-sorbitol, and melibiose. In Biolog GEN III plates, substrates including maltose, cellobiose, sucrose, turanose, d-salicin, *N*-acetyl-d-glucosamine, *α*-d-glucose, d-fructose, d-arabitol, and glycerol are utilized. The major fatty acids are C_16 : 0_ and anteiso-C_15 : 0_. The sole menaquinone is MK-9(H_4_), and the polar lipids are DPG, PI, PIM, one unidentified phosphoglycolipid and two unidentified lipids. The DNA G+C content of the type strain is 71.6 mol%.

The type strain, HLT2-17 ^T^ (=CGMCC 1.11116^T^=NBRC 110443^T^), was isolated from a soil sample collected from the ice tongue surface of Hailuogou Glacier in Sichuan Province, PR China. The GenBank accession number for the 16S rRNA gene sequence reported in this paper is JX949470. The genome sequence has been deposited at DDBJ/ENA/GenBank under the accession SDWW00000000.

## supplementary material

10.1099/ijsem.0.006433 Supplementary Material.

## Abbreviations

ANI: average nucleotide identity
dDDH: digital DNA–DNA hybridization
DPG: diphosphatidylglycerol
ML: maximum-likelihood
NJ: neighbour-joining
PI: phosphatidylinositol
PIM: phosphatidylinositol mannoside
R2A: Reasoner's 2A
T3PKS: type III polyketide synthases

## References

[1] Kim MC, Ju YH, Hwang UA, Liu P, Pak SH (2021). *Pengzhenrongella sicca* gen. nov., sp. nov., a new member of suborder *Micrococcineae* isolated from High Arctic tundra soil. Int J Syst Evol Microbiol.

[2] Parte AC, Sardà Carbasse J, Meier-Kolthoff JP, Reimer LC, Göker M (2020). List of Prokaryotic names with Standing in Nomenclature (LPSN) moves to the DSMZ. Int J Syst Evol Microbiol.

[3] Xie J, Ren L, Wei Z, Peng X, Qin K (2024). *Pengzhenrongella phosphoraccumulans* sp. nov., isolated from high Arctic glacial till, and emended description of the genus *Pengzhenrongella*. Int J Syst Evol Microbiol.

[4] Lane DJ, Stackebrandt E, Goodfellow M (1991). Nucleic Acid Techniques in Bacterial Systematics.

[5] Yoon S-H, Ha S-M, Kwon S, Lim J, Kim Y (2017). Introducing EzBioCloud: a taxonomically united database of 16S rRNA gene sequences and whole-genome assemblies. Int J Syst Evol Microbiol.

[6] Tamura K, Peterson D, Peterson N, Stecher G, Nei M (2011). MEGA5: molecular evolutionary genetics analysis using maximum likelihood, evolutionary distance, and maximum parsimony methods. Mol Biol Evol.

[7] Kim M, Oh HS, Park SC, Chun J (2014). Towards a taxonomic coherence between average nucleotide identity and 16S rRNA gene sequence similarity for species demarcation of prokaryotes. Int J Syst Evol Microbiol.

[8] Bankevich A, Nurk S, Antipov D, Gurevich AA, Dvorkin M (2012). SPAdes: a new genome assembly algorithm and its applications to single-cell sequencing. J Comput Biol.

[9] Chklovski A, Parks DH, Woodcroft BJ, Tyson GW (2023). CheckM2: a rapid, scalable and accurate tool for assessing microbial genome quality using machine learning. Nat Methods.

[10] Seemann T (2014). Prokka: rapid prokaryotic genome annotation. Bioinformatics.

[11] Blin K, Shaw S, Augustijn HE, Reitz ZL, Biermann F (2023). antiSMASH 7.0: new and improved predictions for detection, regulation, chemical structures and visualisation. Nucleic Acids Res.

[12] Pal C, Bengtsson-Palme J, Rensing C, Kristiansson E, Larsson DGJ (2014). BacMet: antibacterial biocide and metal resistance genes database. Nucleic Acids Res.

[13] Meier-Kolthoff JP, Göker M (2019). TYGS is an automated high-throughput platform for state-of-the-art genome-based taxonomy. Nat Commun.

[14] Jain C, Rodriguez-R LM, Phillippy AM, Konstantinidis KT, Aluru S (2018). High throughput ANI analysis of 90K prokaryotic genomes reveals clear species boundaries. Nat Commun.

[15] Na S-I, Kim YO, Yoon S-H, Ha S, Baek I (2018). UBCG: up-to-date bacterial core gene set and pipeline for phylogenomic tree reconstruction. J Microbiol.

[16] Katoh K, Standley DM (2013). MAFFT multiple sequence alignment software version 7: improvements in performance and usability. Mol Biol Evol.

[17] Nguyen L-T, Schmidt HA, von Haeseler A, Minh BQ (2015). IQ-TREE: a fast and effective stochastic algorithm for estimating maximum-likelihood phylogenies. Mol Biol Evol.

[18] Richter M, Rosselló-Móra R (2009). Shifting the genomic gold standard for the prokaryotic species definition. Proc Natl Acad Sci USA.

[19] Wayne LG, Brenner DJ, Colwell RR, Grimont PAD, Kandler O (1987). International Committee on systematic Bacteriology. Report of the *ad hoc* committee on reconciliation of approaches to bacterial systematics. Int J Syst Bacteriol.

[20] Smibert RM, Krieg NR, Gerhardt P, Murray RGE, Wood WA, Krieg NR (1994). Methods for General and Molecular Bacteriology.

[21] Sasser M (1990). Technical Note.

[22] Komagata K, Suzuki K (1988). Lipid and cell-wall analysis in bacterial systematics. Methods Microbiol.

[23] Collins MD, Goodfellow M, Minnikin DE (1985). Chemical Methods in Bacterial Systematics.

[24] Lee C-M, Weon H-Y, Hong S-B, Jeon Y-A, Schumann P (2008). *Cellulomonas aerilata* sp. nov., isolated from an air sample. Int J Syst Evol Microbiol.

