# Supplementary material for: Pengzhenrongella frigida sp. nov., isolated from a glacier
Source: Int J Syst Evol Microbiol. 2024 Jun 19;74(6):006433. doi: 10.1099/ijsem.0.006433 (PMC11261710; doi:10.1099/ijsem.0.006433)
Supplement: Supplementary Material. [file ijsem-74-06433-s001.pdf]

Description of *Pengzhenrongella frigida* sp. nov. isolated from glacier

Qing Liu, Lei-Lei Yang, Yu-Hua Xin*

***Corresponding author:**

Yu-Hua Xin, Email: xinyh@im.ac.cn

Table S1. The number of genes of strains HLT2-17^{T} classified into Cluster of Orthologous Groups (COG) categories.

	No. of genes
Carbohydrate transport and metabolism	422
Transcription	320
General function prediction only	299
Signal transduction mechanisms	296
Amino acid transport and metabolism	249
Coenzyme transport and metabolism	227
Cell wall/membrane/envelope biogenesis	221
Translation, ribosomal structure and biogenesis	215
Inorganic ion transport and metabolism	214
Energy production and conversion	209
Posttranslational modification, protein turnover, chaperones	158
Replication, recombination and repair	148
Lipid transport and metabolism	140
Function unknown	133
Defense mechanisms	107
Nucleotide transport and metabolism	96
Intracellular trafficking, secretion, and vesicular transport	58
Cell cycle control, cell division, chromosome partitioning	54
Cell motility	51
Secondary metabolites biosynthesis, transport and catabolism	49
Mobilome: prophages, transposons	42
Extracellular structures	29
RNA processing and modification	1

Table S2. The whole cell fatty acids composition (%) of HLT2-17^T and the related type strains of the genus *Cellulomonas*.

Strains: 1, HLT2-17^T; 2, *Pengzhenrongella sicca* LRZ-2^T; 3, *C. biazotea* CGMCC 1.1899^T; 4, *C. aerilata* CGMCC 4.7075 ^{T}. Values are percentages of the total fatty acids. tr, traces (less than 1% of the total fatty acids); -, not detected. *, data from Kim *et al.* (2021).

Summed Features are fatty acids that cannot be resolved reliably from another fatty acid using the chromatographic conditions chosen. The MIDI system groups these fatty acids together as one feature with a single percentage of the total. Summed features consist of: 3, $C_{16:1}\omega 7c$ and/or $C_{16:1}\omega 6c$; 8, $C_{18:1}$ $\omega 6c$/$C_{18:1}$ $\omega 7c$.

	1	2*	3	4
**Saturated**				
$C_{14:0}$	2.1	Tr	4.1	5.2
$C_{16:0}$	15.4	1.9	9.3	17.3
$C_{18:0}$	1.1	Tr	1.2	Tr
**Saturated branched**				
iso-$C_{13:0}$	Tr	-	Tr	Tr
iso-$C_{14:0}$	6.2	2.0	8.4	4.5
anteiso-$C_{15:1}$ A	6.8	13.1	10.3	21.4
iso-$C_{15:0}$	1.5	5.2	6.0	Tr
anteiso-$C_{15:0}$	36.6	39.5	40.3	42.3
anteiso-$C_{17:0}$	4.2	11.0	5.5	1.7
iso-$C_{16:0}$	5.3	23.9	6.8	3.7
**Unsaturated**				
$C_{16:1}$ $\omega 9c$	2.1	-	-	-
$C_{17:1}$ $\omega 8c$	1.1	-	-	Tr
$C_{18:1}$ $\omega 9c$	1.5	Tr	Tr	Tr
anteiso-$C_{17:1}$ $\omega 9c$	Tr	-	-	-
**Hydroxy**				
$C_{15:0}$ 3-OH	-	-	1.1	Tr
**Summed feature***				
summed feature 3	8.4	-	-	Tr
summed feature 8	1.1	-	-	Tr

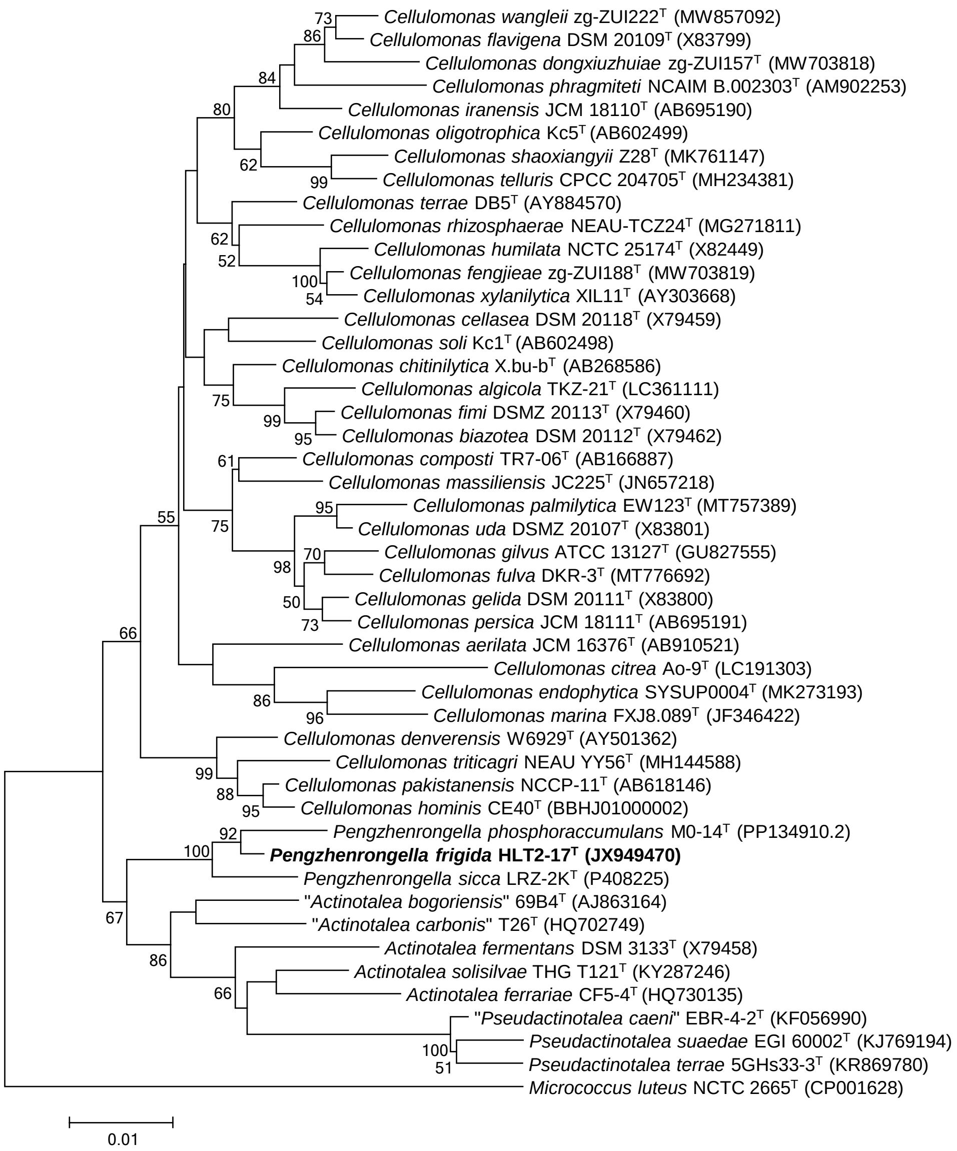

Fig. S1. Phylogenetic tree of strain HLT2-17^T and related strains based on the 16S rRNA gene sequence comparisons using the NJ method. GenBank accession numbers of the 16S rRNA gene sequences are given in parentheses. Bootstrap values (>50 %) based on 1,000 replicates are shown at the branch nodes. Bar, 0.01 substitutions per nucleotide positions.

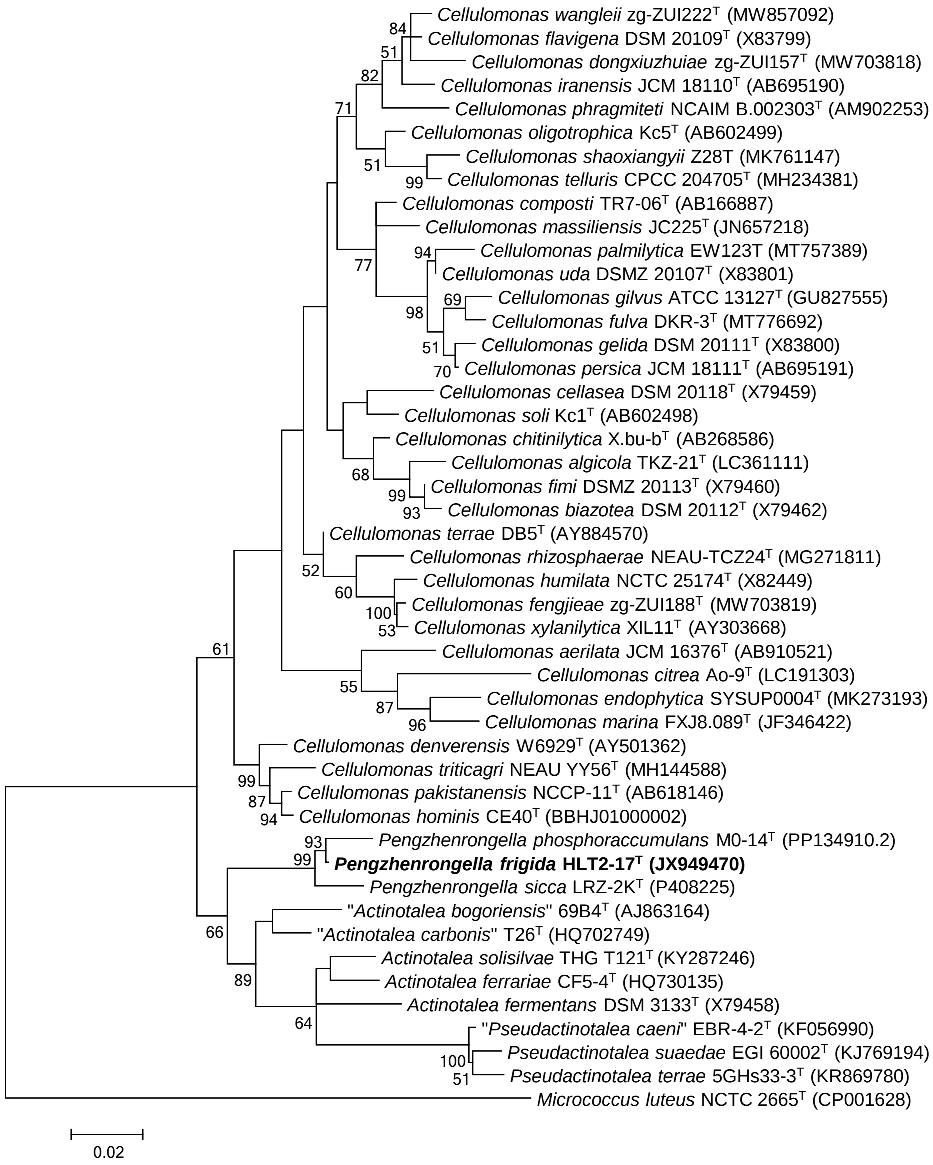

Fig. S2. Phylogenetic tree of strain HLT2-17^{T} and related strains based on the 16S rRNA gene sequence comparisons using the ML method. GenBank accession numbers of the 16S rRNA gene sequences are given in parentheses. Bootstrap values (>50 %) based on 1,000 replicates are shown at the branch nodes. Bar, 0.01 substitutions per nucleotide positions.

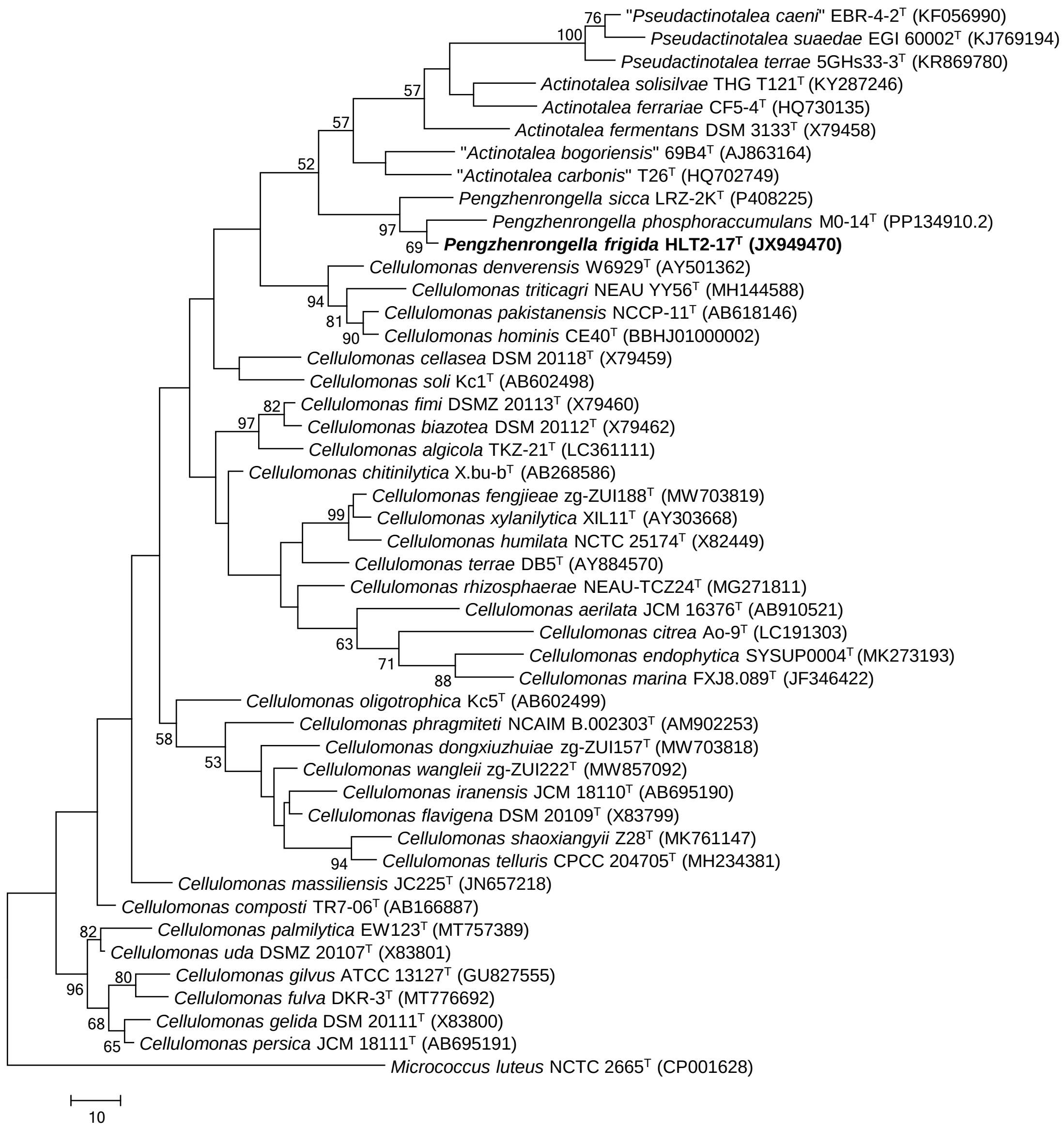

Fig. S3. Phylogenetic tree of strain HLT2-17^{T} and related strains based on the 16S rRNA gene sequence comparisons using the MP method. GenBank accession numbers of the 16S rRNA gene sequences are given in parentheses. Bootstrap values (>50 %) based on 1,000 replicates are shown at the branch nodes. The tree is drawn to scale , with branch lengths calculated using the average pathway and are in the units of the number of changes over the whole sequence .

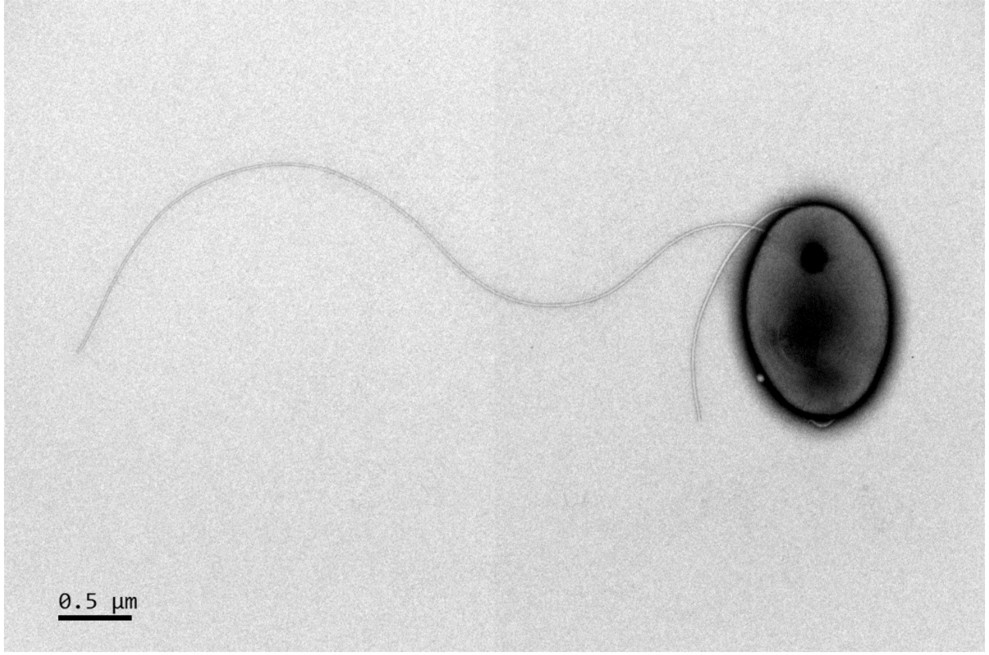

Fig. S4. Transmission electron micrograph of strain HLT2-17^{T} grown at 20°C on R 2A agar. Bar, 0.5 µm.

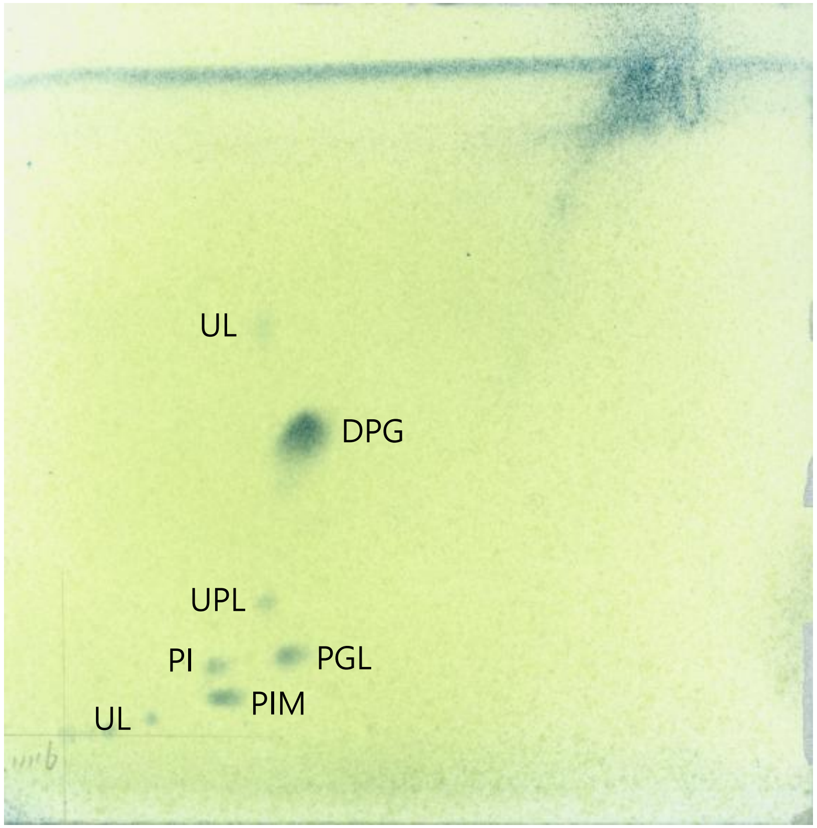

Fig. S5. Two-dimensional TLC of the polar lipids of strain HLT2-17^{T} after spraying with 5 % ethanolic molybdatophosphoric acid.

DPG: diphosphatidylglycerol; PI: phosphatidylinositol; PIM: phosphatidy linositol mannoside; PGL unknown phosphoglycolipid; UL: unidentified lipid.
